# Advancements in Biomarkers for Early Detection and Risk Stratification of Cardiovascular Diseases—A Literature Review

**DOI:** 10.1002/hsr2.70878

**Published:** 2025-05-26

**Authors:** Abubakar Nazir, Awais Nazir, Usama Afzaal, Shafaq Aman, Safi ur Rehman Sadiq, Ozoemena Z. Akah, Muhammad Shah Wali Jamal, Syed Zawahir Hassan

**Affiliations:** ^1^ Oli Health Magazine Organization, Research and Education Kigali Rwanda; ^2^ Department of Medicine King Edward Medical University Lahore Pakistan; ^3^ St John of God Midland Hospitals Australia; ^4^ Mountain View Hospital Las Vegas Nevada USA; ^5^ Division of Cardiovascular Prevention Houston Methodist DeBakey Heart & Vascular Center Houston USA

**Keywords:** artificial intelligence, cardiovascular biomarkers, clinical validation, early detection, genomics, metabolomics, microRNAs, multi‐omic approaches, personalized medicine, precision medicine, proteomics, risk stratification

## Abstract

**Introduction:**

CVDs is a leading cause of morbidity, mortality, and healthcare expenditure worldwide. Identifying individuals at risk or in the incipient stages of disease is instrumental in enabling timely interventions, preventive measures, and tailored treatment regimens. The landscape of CVDs is complicated by their heterogeneity, encompassing a spectrum of conditions such as coronary artery disease, heart failure, arrhythmias, and valvular disorders. In recent years, the integration of biomarkers into cardiovascular medicine has emerged as a paradigm‐shifting approach with the potential to revolutionize early detection and risk stratification. By synthesizing a multitude of studies, we aim to provide a comprehensive resource that illuminates the transformative potential of biomarkers in ushering in a new era of precision cardiovascular medicine.

**Aim:**

To identify the biomarkers for the detection and diagnosis of CVDs.

**Materials and Methods:**

This review examines key studies from 2015 to the present that investigate the impact of cardiac biomarkers on cardiovascular outcomes. Data were gathered from PubMed, Cochrane Library, and Embase to ensure a comprehensive analysis. The review focuses on various cardiac biomarkers, assessing their levels and changes in relation to cardiovascular health, with special emphasis on advanced biomarkers such as proteomic and metabolomic markers in cardiovascular disease (CVD) diagnosis. Peer‐reviewed studies published in English that evaluated the diagnostic, prognostic, or therapeutic role of cardiac biomarkers were included, with priority given to clinical trials, cohort studies, systematic reviews, and meta‐analyses providing quantitative biomarker data. Studies unrelated to cardiac biomarkers, case reports, editorials, conference abstracts, and those with small sample sizes or insufficient methodological rigor were excluded. The review also accounts for potential confounding factors and research limitations, ensuring a balanced assessment of the literature. By synthesizing data from academic papers, clinical reports, and research articles, this study provides a comprehensive evaluation of the evolving role of cardiac biomarkers in CVD diagnosis and risk stratification.

**Results:**

Biomarkers play a pivotal role in cardiovascular disease risk prediction, diagnosis, and treatment by providing dynamic biological insights. High‐sensitivity cardiac troponins (hs‐cTn) enhance myocardial injury detection, while circulating microRNAs (miR‐208, miR‐499) serve as early indicators of myocardial infarction and heart failure. Lipoprotein(a) [Lp(a)] predicts long‐term cardiovascular risk, and inflammatory biomarkers such as C‐reactive protein (CRP) and interleukin‐6 (IL‐6) are linked to adverse outcomes. Multi‐biomarker panels, such as hs‐cTn with B‐type natriuretic peptide (BNP), improve heart failure prognosis, while metabolomic profiling enables precision medicine. Additionally, biomarkers like BNP and NT‐proBNP facilitate real‐time therapeutic monitoring. These findings underscore the critical role of biomarkers in refining risk stratification, improving diagnostic accuracy, and enabling personalized treatment strategies in cardiovascular medicine.

**Conclusion:**

The advancement of cardiovascular biomarkers has significantly enhanced early detection, risk stratification, and personalized treatment. Emerging biomarkers, including genetic variants, metabolomics, microRNAs, and imaging‐based markers, provide deeper insights into disease mechanisms. Integrating multi‐omic approaches with artificial intelligence may further refine predictive accuracy and therapeutic decision‐making. However, clinical translation requires rigorous validation through large‐scale, multicenter studies to ensure reliability and applicability across diverse populations. Standardization, cost‐effectiveness assessments, and the development of biomarker panels are essential for clinical adoption. Future research should focus on bridging discovery and implementation, advancing precision medicine to improve cardiovascular outcomes.

## 1 Introduction

Cardiovascular diseases (CVDs) stand as a formidable global health challenge, exacting an enormous toll on societies in terms of both human suffering and economic burden. With their intricate pathophysiology and often asymptomatic early stages, CVDs continue to be a leading cause of morbidity, mortality, and healthcare expenditure worldwide [1]. According to the World Health Organization (WHO), an estimated 17.9 million deaths were attributed to CVDs in 2019 alone, representing 31% of all global deaths. This staggering statistic underscores the urgent need to employ strategies that facilitate early detection and precise risk stratification to curtail the CVD epidemic [1].

Early detection and precise risk stratification are crucial in mitigating the impact of cardiovascular diseases (CVDs). Identifying individuals at risk or in early stages of disease allows for timely interventions, personalized treatments, and preventive measures [2]. These proactive approaches not only enhance patient care and quality of life but also have the potential to reduce healthcare system burdens and long‐term costs. By preemptively identifying high‐risk individuals, healthcare providers can implement interventions that slow or halt disease progression, thereby preventing serious events such as heart attacks, strokes, and heart failure. The heterogeneous nature of CVDs, encompassing conditions like coronary artery disease, heart failure, arrhythmias, and valvular disorders, poses challenges in developing strategies that enable early detection and differentiate between varied manifestations. Biomarkers, including proteins, lipids, nucleic acids, and metabolites, have emerged as pivotal tools in cardiovascular medicine. They offer insights into physiological processes and disease mechanisms, aiding in the early detection of deviations from normal function and guiding targeted therapeutic approaches. Thus, biomarkers represent a transformative approach in cardiovascular healthcare by enabling more precise diagnosis and intervention strategies [3, 4, 5].

**Figure 1:**
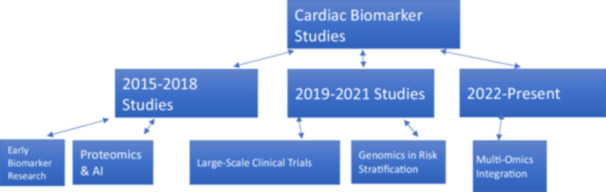
A tree diagram summarizing previous studies.

This review underscores the multifaceted role of biomarkers in revolutionizing the management of cardiovascular diseases (CVD). Beyond enhancing diagnostic accuracy, biomarkers enrich traditional risk prediction models by integrating dynamic biological data, thereby refining prognostic assessments. They also facilitate personalized treatment strategies by enabling targeted therapies tailored to an individual's molecular profile. Biomarkers serve crucial roles in monitoring treatment efficacy, enabling clinicians to make timely adjustments. The article explores a diverse array of biomolecules—from established markers like high‐sensitivity cardiac troponins and brain natriuretic peptides to emerging candidates in genomics, proteomics, and metabolomics. This comprehensive analysis not only highlights their diagnostic capabilities but also their potential to forecast disease progression and therapeutic outcomes. Ultimately, biomarkers are poised to transform cardiovascular medicine by advancing precision healthcare approaches (Figure 1).

## 2 Traditional Biomarkers for Cardiovascular Disease Detection and Risk Assessment and Their Limitations

Traditional biomarkers have played a foundational role in cardiovascular disease (CVD) detection and risk assessment, forming the basis of diagnostic and prognostic strategies for decades. These biomarkers encompass a range of physiological and biochemical indicators, including cholesterol levels, C‐reactive protein (CRP), and others. While they have provided valuable insights into CVD pathophysiology, their limitations underscore the need for novel biomarkers to enhance accuracy and predictive value. Cholesterol, a lipid molecule, has been a central focus of cardiovascular risk assessment due to its role in atherosclerosis, a condition characterized by the buildup of fatty deposits in arterial walls. Low‐density lipoprotein cholesterol (LDL‐C), often referred to as “bad” cholesterol, has been a primary target for risk assessment and intervention. Elevated LDL‐C levels are associated with an increased risk of atherosclerosis and subsequent cardiovascular events. However, the specificity of LDL‐C as a standalone biomarker is limited. Individuals with normal LDL‐C levels can still develop CVD, as other factors such as genetics, inflammation, and endothelial dysfunction contribute to disease progression [3, 5, 6]. C‐reactive protein (CRP) is an acute‐phase reactant produced by the liver in response to inflammation. High‐sensitivity CRP (hsCRP) has gained prominence as a marker of cardiovascular risk, particularly due to the role of chronic inflammation in atherosclerosis. Elevated hsCRP levels have been associated with increased cardiovascular risk. However, CRP's limitations are evident in its lack of specificity. Infections, injuries, and other inflammatory conditions can also elevate CRP levels, potentially leading to false‐positive results. As a result, while hsCRP offers insights into inflammation's role in CVD, it falls short in accurately predicting individual risk [5, 6].

### 2.1 Limitations and Challenges Associated With Traditional Biomarkers

Both cholesterol and CRP, as well as other traditional biomarkers, lack the specificity needed to reliably predict individual cardiovascular risk. This can lead to misclassifying individuals who may not actually be at high risk. Cardiovascular diseases arise from intricate interactions between genetic, lifestyle, and environmental factors. Traditional biomarkers may not capture the multifaceted nature of these diseases, potentially resulting in incomplete risk assessments [6, 7]. Traditional biomarkers often become abnormal only when significant disease progression has occurred. This limitation prevents timely interventions that could potentially prevent or slow disease progression. Different individuals may respond differently to similar levels of traditional biomarkers. Genetic, lifestyle, and other factors can modify the impact of these biomarkers on an individual's risk. Traditional biomarker levels can fluctuate over time due to various factors, making it challenging to use them as reliable long‐term predictors of risk [7].

### 2.2 Need for Novel Biomarkers

**Figure 2:**
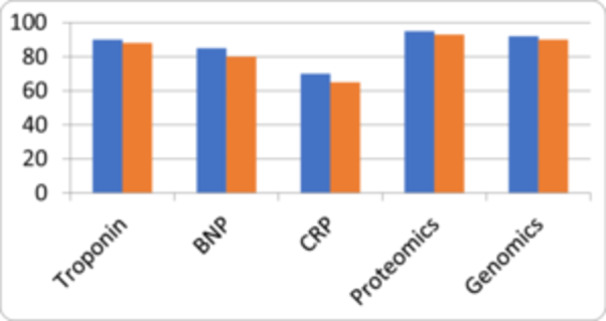
Bar chart comparing biomarker sensitivity & specificity (Troponin (90/88%), BNP (85/80%), CRP (70/65%), Proteomics (95/93%), and Genomics (92/90%) demonstrate varying sensitivity and specificity in cardiovascular disease detection, with proteomics showing the highest accuracy.

The shortcomings of traditional biomarkers have prompted researchers to seek novel indicators that offer greater accuracy, specificity, and predictive value for cardiovascular risk assessment (Figure 2). These emerging biomarkers span various categories including genetic markers, metabolomics, microRNAs and image based biomarkers. Genetic studies, including genome‐wide association studies (GWAS), have identified specific genetic variants associated with increased cardiovascular risk. These markers provide insights into an individual's genetic predisposition to CVD and can contribute to more personalized risk assessments [8, 9]. MicroRNAs are small RNA molecules that regulate gene expression. Certain microRNAs are dysregulated in cardiovascular diseases, reflecting specific pathophysiological processes. These molecules show promise as potential biomarkers due to their specificity and potential to indicate disease progression [9]. Metabolomics involves profiling a broad range of metabolites in biological samples. Altered metabolite patterns can reveal insights into disease states and metabolic pathways that contribute to CVD. Metabolomics offers a comprehensive view of an individual's physiological state. Advanced imaging techniques, such as coronary artery calcium scoring and carotid intima‐media thickness measurements, provide direct visualization of structural and functional changes associated with atherosclerosis and CVD. These imaging biomarkers add a layer of precision to risk assessment. Traditional biomarkers like cholesterol and CRP have been instrumental in shaping our understanding of cardiovascular risk. However, their limitations have highlighted the need for novel biomarkers that offer enhanced accuracy and predictive value. The ongoing exploration of genetic markers, microRNAs, metabolites, and imaging‐based indicators represents a promising frontier in cardiovascular medicine, paving the way for more precise risk assessment, early intervention, and improved patient outcomes.

## 3 Emerging Biomarkers for Early Detection and Risk Stratification

Among the most common causes of mortality and morbidity in developed as well as developing countries coronary artery disease and acute myocardial infarction (AMI) are still gone undiagnosed in their early stages [10]. In US death rate has been decreasing recently that may be attributed to early diagnosis and efficient revascularization techniques. It may be decreased further if new biomarkers are developed that can detect coronary artery disease as early as possible [11]. New biomarkers are being discovered and lots of clinical trials are being conducted on them to determine their effectiveness in early diagnosis, risk stratification and efficient management of the patients. In young age, the process of atherosclerosis is initiated and timed screening may ensue in outcomes that are in our best interests [12]. Three characteristics of biomarkers that must be present in it beside good sensitivity and specificity are [10]:

1. Having a predictable nature
2. Having a good accessible site
3. Having a reliable method of detection.

Still their clinical uses and precision must be justified before inculcating them in clinical practice. Biomarkers associated with myocardial injury, instability and rupturing of plaques, inflammation, activation of platelets, myocardial stress, microRNAs and myocardial stress are being under investigation for further implementation. Cardiac troponins, high sensitive cardiac troponins (hs‐cTn), Heart‐type fatty acid binding protein, High‐sensitivity C‐reactive protein (hsCRP), Pregnancy‐associated plasma protein‐A (PAPP‐A), Matrix metalloproteinases (MMPs), Lipoprotein‐associated phospholipase A2 (Lp‐PLA2), Natriuretic peptides, and ST2 are some biomarkers that are still under investigation for their potential clinical benefits [13] (Table 1).

**Table 1:** Shows the biomarkers and cardiovascular events with which they are associated [13].

Biomarkers	Cardiovascular events with which they are associated
Cardiac troponin, High‐sensitivity assays for cardiac troponin, Heart‐type fatty acid binding protein	Diagnosing myocardial infarction in its early symptomology
Growth differentiation factor‐15, Fibrinogen, Uric acid, high‐sensitivity C‐reactive protein	Predicting MI and Death following it
Pregnancy‐associated plasma protein A, matrix metalloproteinases, myeloperoxidase	Risk of acute coronary syndrome
Secretory phospholipase A2, lipoprotein‐associated phospholipase A2	Incidence and recurrence of cardiovascular events
Mid‐regional‐pro‐adrenomedullin, copeptin, elevated natriuretic peptides, ST2, endothelin‐1, galectin‐3	Heart failure and death following an MI and stratification of risk associated with it

Now‐a‐days cardiac troponins are included in clinical practice for early detection of acute myocardial infarction. The two troponins that are measured to quantify the amount of damage of cardiomyocytes are cTnT and cTnI [14]. Their concentrations in blood will be directly to the amount of injury to cardiomyocytes. There is no evidence that which type of cardiac troponin is better for the diagnosis of myocardial infarction [15]. They have the greatest sensitivity and specificity in diagnosis AMI when clinical symptomology is evident than myoglobin and creatine kinase (CK) along with its isoenzyme MB (CK‐MB). In clinical symptomatic patients above 99th percentile of normal individuals indicates AMI [16]. Old methods of analysis have a lower sensitivity at an early stage of AMI and require continuous sampling over 6 to 9 h to reach diagnosis [17]. Hs‐cTn have solved this problem and have increased the accuracy of diagnosis at presentation and subsequently decreased the time interval for next measurement. It can even detect the troponins in clinical stable patients and healthy individuals. Cardiac troponins usually rise in first hour following AMI if using high‐sensitive assays and this lead to greater diagnostic accuracy in diagnosing early AMI and reducing healthcare cost [13, 14]. Finding troponins in healthy individuals and developing a more reliable definition are the two distinct features of high sensitive assays as compared to conventional assays [18]. So, finding a baseline value of hs‐cTn has lead to risk stratification in patients with CAD and diabetes for all cause death, stroke, AMI, and heart failure [19].

MicroRNAs (miRNAs), small noncoding RNA molecules about 17–25 nucleotides long, are emerging as potential biomarkers for coronary artery disease (CAD). They regulate gene expression post‐transcriptionally by inhibiting translation or causing mRNA degradation. Over 100 miRNAs have been identified circulating in plasma and serum, categorized into vesicle‐associated and non‐vesicle forms. In the heart, miRNAs like miR‐26a, miR‐1, miR‐133, miR‐126‐3p, let‐7, and miR‐30c are prevalent. Studies indicate that levels of specific miRNAs, such as miR‐208a, miR‐133a, miR‐92a, miR‐126, miR‐17, and miR‐155, vary in CAD patients, distinguishing between stable and unstable angina and acute myocardial infarction (AMI) [10, 11, 12, 13, 14, 15, 16]. Elevated miRNA levels like miR‐17‐5p correlate with CAD severity, while decreased levels of miR‐16 have been noted. Different miRNAs are implicated in various stages of atherosclerosis and are linked to conditions like type 2 diabetes mellitus, influencing cardiovascular disease development. MiRNAs such as miR‐9, miR‐30, miR‐126, miR‐1, miR‐15a, miR‐133a, miR‐342, miR‐210, miR‐92a, and miR‐155 show promise as biomarkers for risk stratification and noninvasive CAD diagnosis, particularly in patients with type 2 diabetes. Despite their potential, challenges such as reproducibility, cost‐effectiveness, standardization of detection methods, and sample size limitations need addressing before miRNAs can be widely adopted in clinical practice. Nevertheless, miRNAs have demonstrated sensitivity and specificity as markers for CAD in various studies [10, 11, 12, 13, 14, 15, 16, 17, 18, 19, 20, 21, 22, 23, 24, 25, 26, 27]. Imaging techniques such as carotid intima‐media thickness (CIMT) measured by ultrasound and coronary artery calcium score (CACS) assessed by CT scan are under study for personalized risk stratification in cardiovascular disease. CIMT is sensitive for detecting subclinical atherosclerosis and is considered a potential predictor of adverse cardiovascular events. In contrast, CACS, which detects calcification—an advanced stage of atherosclerosis—has lower predictability compared to CIMT. Many individuals with subclinical atherosclerosis detected by CIMT have zero CACS, highlighting the difference in their sensitivity to early disease stages. Both CIMT and CACS are recommended for CAD risk stratification, with elevated CACS indicating higher risk and zero CACS suggesting lower or intermediate risk. Screening with these techniques not only identifies high‐risk patients but also reveals subclinical disease, particularly in those with low Framingham risk scores and zero CACS, informing strategies for primary prevention of CAD [28].

Beside these biomarkers and imaging techniques bone biomarkers such as alkaline phosphatase, osteoprotegerin, and osteopontin have been found to aid in diagnosis and most importantly in prognosis of CAD [29]. In addition, Triglyceride‐glucose index is found to be a valuable index for early intervention and risk stratification in patients of premature coronary artery disease [30]. There is also evidence that in nondiabetic patients plasma biomarkers such as high‐sensitivity C‐reactive protein (hsCRP) and the ratio of total cholesterol (TC) to high‐density lipoprotein cholesterol (HDL‐C) remained high in patients with adverse cardiovascular events and thus anticipate the forthcoming cardiovascular events and so they may be used in clinical risk stratification [31].

No doubt that usage of biological biomarkers has increased the accuracy of diagnosis and risk stratification in CAD. But to our dismay the sensitivity and specificity all of them except in case of troponins and CK‐MB remain poor [32]. Studies conducted by Zampetaki, et al. and Bye, et al. have shown that the prediction power of the tradition risk factor model Framingham score system improved when it combined with miRNA profile but not a single miRNA level suggests an increased risk of AMI when used alone [33, 34]. Nowadays, multimarker analysis has been suggested as it increases the specificity and sensitivity of the prognostic tools. So, when multiple biomarkers are combined together, they may be useful in predicting cardiovascular events as compared to when they are used alone. Zagidullin found that combined multimarker analysis in two biomarker combinations (NT‐proBNP+Ptx‐3) have been found to be most accurate in predicting 2‐year cardiovascular mortality after STEMI. He also suggested that the most accurate combo was a three‐biomarker model (NT‐proBNP+Ptx‐3 + ST2) in predicting 2‐year cardiovascular mortality [35].

Recent genetic studies have identified specific genetic loci and genes that independently increase the likelihood of cardiovascular disease (CVD) and its risk factors from birth, with interactions with the environment potentially contributing to disease development. These genetic factors are now being considered in the development of personalized medications. Single‐nucleotide polymorphisms (SNPs), which involve a single nucleotide base change in DNA, have been associated with coronary artery disease (CAD). SNPs can alter gene expression, affecting the development of CAD and its severity, although outcomes can vary widely among individuals with the same polymorphisms. Genetic biomarkers, particularly SNPs, are more specific compared to nongenetic markers, guiding targeted therapies even in asymptomatic individuals. They play crucial roles in CAD diagnosis, prognostic evaluation, risk stratification, pharmacokinetics, pharmacodynamics, and personalized medicine. Genetic risk scores (GRS), calculated from genotype data, incorporate multiple SNPs to enhance prognostic accuracy. The inclusion of additional SNPs in GRS improves its predictive power independently of family history, particularly beneficial in younger patients. Despite the potential of GRS, its effectiveness varies across studies due to differences in SNP selection and ethnic diversity. Further functional studies are necessary to refine GRS for precise risk stratification and prognostic evaluation in modern cardiology, aiming to reduce CAD risk and enhance lifespan and quality of life [36, 37, 38] (Table 2).

**Table 2:** Compared results with the previous work.

Biomarkers analyzed	Key findings	Limitations	How this review adds value
Traditional biomarkers (e.g., troponins, natriuretic peptides)	Emphasized the diagnostic value of traditional biomarkers in acute settings but noted limitations in long‐term risk prediction [7, 38].	Focused primarily on established biomarkers; limited discussion on novel biomarkers and emerging technologies.	This review expands upon this by incorporating recent advancements in biomarker discovery, including genetic and proteomic markers, providing a more comprehensive overview of current and potential future biomarkers [38].
Traditional biomarkers (e.g., troponins, natriuretic peptides)	Discussed the role of traditional biomarkers in diagnosing acute coronary syndromes and heart failure.	Did not address emerging biomarkers or novel detection methods.	This review includes recent studies on novel biomarkers such as microRNAs and exosomal proteins, highlighting their potential in early detection and risk stratification [10, 13, 14].
LDL cholesterol, C‐reactive protein (CRP), lipoprotein(a)	Introduced a “three‐pronged” blood test measuring LDL cholesterol, CRP, and lipoprotein(a) to predict heart disease risk up to 30 years in advance. Found that high levels of these biomarkers significantly increased the risk of cardiovascular events [15].	Study focused on a specific population (American women); findings may not be generalizable to other groups.	This review contextualizes these findings within a broader spectrum of biomarkers and discusses the integration of such multi‐marker approaches into clinical practice [15].
LDL cholesterol, lipoprotein(a), high‐sensitivity CRP	Demonstrated that testing for three biomarkers can predict the risk of heart attack, stroke, or other cardiovascular issues 30 years later. Highlighted the need for routine screening of these biomarkers early in life to aid in preventive measures against cardiovascular diseases.	Focused exclusively on women; results may not apply to men.	This review discusses the applicability of these biomarkers across different populations and genders, providing a more inclusive perspective [17].
Genetic risk scores	Introduced a genetic test capable of predicting the risk of common diseases, including cardiovascular diseases, by generating genetic risk scores using machine learning. Aimed to enable personalized, preventative healthcare by identifying high‐risk individuals early.	Implementation and accessibility of genetic testing in routine clinical practice remain challenges.	This review explores the potential of integrating genetic risk scores with traditional biomarkers to enhance early detection and personalized risk stratification [36].

## 4 Technological Advances and Personalized Medicine and Proteomic and Metabolomic Biomarkers for Risk Stratification

A biomarker indicates a special state of a body that can be physiological, pathological or pharmalogical. Novel biomarkers are very much important because they aid in diagnosis, prognosis, and precision medicine as well as response to a particular therapy. Nowadays, advancement in fields of proteomics, genomics, and metabolomics have been made for the discovery of new biomarkers. This involves the use of techniques such as mass spectrometry, liquid chromatography–mass spectrometry (LC‐MS), 2‐gel electrophoresis (2DE) united to matrix‐assisted laser desorption time‐of‐flight mass spectrometry (MALDI‐TOF) tandem mass spectrometry, two‐dimension liquid chromatography, aptamer‐based assays, Proximity Extension Assays (Olink), multiplex immunoassays microarray and sequencing [39, 40].

Proteomics is the study of proteins, encompassing their composition, interactions, structures, functions, and cellular activities. It involves a meticulously regulated multi‐step process to prevent nonorganic factors that could alter protein expression and interactions. Sample acquisition is crucial, particularly in solubilizing proteins and mitigating interference from molecules like lipids. Plasma, containing thousands of proteins, is a key source for biomarker investigation, though many protein‐disease relationships remain undiscovered, particularly in diseases like coronary artery disease (CAD). Plasma is convenient but lacks specificity, as it may contain proteins from various tissues. Tissue‐based analysis is considered the gold standard for diagnosis due to its reliability. Recent studies have identified specific proteins in plasma associated with conditions such as myocardial infarction and left ventricular dysfunction, highlighting their potential as biomarkers. Combining protein panels with established clinical risk scores enhances the accuracy of risk stratification compared to using scores alone, underscoring proteomics' role in advancing diagnostic and prognostic capabilities in cardiovascular medicine [41, 42, 43].

Genomics involves the study of DNA sequencing, chimeric DNA, and bioinformatics to analyze and understand the structure and function of an organism's entire set of DNA, known as its genome. In clinical medicine, genomics focuses on the functional genome, exploring individual genes from their expression to their roles in biological functions. Genome Wide Association Studies (GWAS) are crucial in identifying genetic variants like SNPs (single nucleotide polymorphisms) associated with conditions such as coronary artery disease (CAD). Techniques employed include genome annotation, mutagenesis, RNA interference (RNAi), SNP analysis, and genetic interaction mapping. The National Human Genome Research Institute (NHGRI) plays a key role in storing and exploring the human genome and its implications for various diseases. Family and twin studies indicate that a substantial portion (40%–50%) of CAD risk is heritable. While GWAS have identified around 31 loci linked to CAD, these explain less than 10% of its heritability. Recent research has identified approximately 45 loci specifically associated with CAD in South Asian and European populations, highlighting ongoing efforts to understand the genetic underpinnings of cardiovascular diseases [44, 45, 46].

Metabolomics is the comprehensive study of all metabolites present in biological samples or organisms, encompassing over 19,000 small molecules derived from both endogenous enzymatic processes and exogenous sources like food, drugs, microbiota, and the environment. This field differs from proteomics and genomics in its broad scope and focus on understanding how metabolite levels change in response to external factors and disease. By integrating metabolomics with other omics disciplines, new biomarkers associated with cardiovascular disease can be identified and combined with existing genetic and clinical data to enhance risk stratification, diagnosis, and prognosis. Metabolomics plays a crucial role in early disease detection by identifying molecular changes that precede clinical symptoms, offering insights into disease mechanisms such as insulin resistance and obesity leading to type II diabetes mellitus. Specific metabolites like branched chain amino acids (BCAA) and markers such as trimethylamine N‐oxide (TMAO) have been linked to conditions like coronary artery disease and myocardial infarction, detecting abnormalities shortly after the onset of symptoms. Metabolomics also reveals associations between metabolite levels and cardiovascular events, providing predictive markers for disease progression and patient outcomes [47, 48, 49, 50, 51, 52].

The concept of understanding human physiology extends beyond studying individual molecules like DNA, RNA, proteins, and metabolites, to encompassing environmental and other factors. This holistic approach is known as “multi‐omics,” which aims to elucidate the relationships between these molecules and their roles in disease pathology, diagnosis, and prognosis. For instance, genetic causes of diseases such as familial hypercholesterolemia involve complex interactions that go beyond lipid‐lowering drugs alone. External factors like diet can influence genetic expression, impacting disease risk. Multiple biomarkers have been identified for coronary artery disease (CAD), enhancing risk stratification. Integrating vast amounts of data requires digital tools such as algorithms and risk calculators that incorporate nuclear, cellular, and enzymatic components. Multi‐omics is particularly valuable for noncoding genes and understanding the downstream effects of genetic variants. Studies have linked genes like PCSK9 and loci such as 9p21 to CAD risk through their influence on lipid levels and vascular health. Epigenetic modifications such as DNA methylation contribute to CAD risk by affecting processes like atherosclerosis. This integrative approach, spanning genomics, transcriptomics, proteomics, and other omics fields, uses software and artificial intelligence to predict and manage CAD risk effectively [47, 53, 54].

Personalized medicine is a new field in which a specific treatment in addition to all tailored clinical decisions is given to a particular person. It combines the information from various omics to identify the culprit step and give the treatment according to that step. Biomarkers are very much important as they can indicate that specific disturbed steps even at earlier stages. In this era, genomics and other biomarkers improves the diagnosis and therapeutic effects of disease through personalized medicine. It is often used in genetic testing, lifestyle changes, and risk prevention. It is a multidisciplinary approach in which all aspects of an individual are explored before any interpretation and intervention. For example, long QT syndromes (LQTS) can be used for risk probability as 12 different alleles are found at its loci that necessities the genetic testing of the individuals. Beta‐blockers can be used in case of LQTS 1 but not in LQTS2 or LQTS 3. So, personalized medicine is focused on analyzing all the particular factors for the CAD and identifying those particular factors that should be addressed in that particular individuals. For example, when we are dealing with familial hypercholesterolemia, we have to led a specific pathway in dealing with rather than following a traditional way in case of others because diagnosis, prognosis and therapeutics are all different in this case. Finally, this all depends on particular biomarkers that can indicate a specific state at which a specific treatment can be given to a specific person [55, 56].

### 4.1 Strengths of Biomarkers

Biomarkers have significantly enhanced the early detection, risk stratification, and management of cardiovascular diseases by providing objective, quantifiable indicators of disease presence and progression. One of their key advantages is their high sensitivity and specificity, particularly in acute conditions. For instance, cardiac troponins (cTnI, cTnT) have become the gold standard for diagnosing myocardial infarction, while natriuretic peptides (BNP, NT‐proBNP) are widely used to assess heart failure severity [32, 33, 34, 35, 36, 42, 43, 54]. In addition to their diagnostic value, many biomarkers allow for the detection of subclinical disease. High‐sensitivity C‐reactive protein (hs‐CRP) serves as an early indicator of vascular inflammation and is associated with an increased risk of atherosclerosis, while microRNAs and circulating endothelial cells show promise in identifying cardiovascular pathology before structural changes occur [50, 53].

Beyond diagnosis, biomarkers play a crucial role in risk stratification and prognosis. Biomarkers such as galectin‐3 and soluble ST2 (sST2) provide insight into cardiac remodeling and heart failure progression, helping clinicians tailor treatment strategies [29, 30, 31, 32, 33, 34, 44]. Similarly, lipoprotein(a) [Lp(a)] has emerged as an independent predictor of atherosclerotic cardiovascular disease, allowing for more personalized risk assessment, particularly in individuals with a family history of premature coronary artery disease. Another major advantage of biomarkers is their noninvasive nature, as most can be measured through routine blood tests, reducing the need for invasive procedures. Furthermore, advances in molecular profiling have expanded their role in personalized medicine. For example, genetic markers such as CYP2C19 polymorphisms guide antiplatelet therapy selection, ensuring optimal treatment responses in patients with acute coronary syndrome [24, 27, 31, 39].

### 4.2 Limitations of Biomarkers

Despite their numerous advantages, biomarkers also have limitations that impact their clinical utility. One of the most significant challenges is the lack of sufficient validation for many emerging biomarkers. While traditional biomarkers like troponins and BNP are well‐established, newer biomarkers such as exosomal RNA, metabolomic signatures, and proteomic panels require large‐scale validation studies before they can be integrated into clinical practice [43, 47]. Another limitation is the variability of biomarker levels due to individual patient factors. Age, sex, renal function, and other comorbidities can influence biomarker concentrations, leading to potential misinterpretation. For example, BNP levels may be elevated in patients with chronic kidney disease even in the absence of heart failure, which can lead to diagnostic uncertainty. Additionally, differences in assay techniques and lack of standardization across laboratories can result in variations in biomarker measurement, impacting their reliability [32, 35, 38].

Another major concern is the potential for false positives and overdiagnosis. Biomarkers such as hs‐CRP, while useful for assessing cardiovascular risk, lack disease specificity and can be elevated in conditions unrelated to cardiovascular disease, such as infections and autoimmune disorders. This can lead to unnecessary testing and treatment, increasing healthcare costs without clear clinical benefit. The cost and accessibility of biomarker testing further limit their widespread adoption, particularly in resource‐limited settings [43, 46]. Advanced genomic and proteomic biomarker tests, while promising, remain expensive and are not universally covered by insurance, restricting their availability. Lastly, the regulatory approval process for new biomarkers is complex and time‐consuming, delaying their transition from research to clinical use. The integration of multi‐marker panels and artificial intelligence‐driven risk prediction models also faces challenges in implementation, requiring extensive validation and physician training [49, 52].

While biomarkers have transformed cardiovascular disease management, their full potential is yet to be realized due to challenges in validation, standardization, cost, and clinical implementation. Future research should focus on improving the specificity and reliability of biomarker‐based tests, enhancing accessibility, and integrating novel biomarkers into comprehensive risk prediction models. Addressing these limitations will be essential for maximizing the clinical impact of biomarkers in cardiovascular medicine.

### 4.3 Novel Techniques and Highlight on the Clinical Significance

The field of cardiovascular biomarkers has evolved significantly, with emerging technologies offering greater sensitivity and predictive power than traditional biomarkers like cardiac troponins (cTn), B‐type natriuretic peptide (BNP), and C‐reactive protein (CRP). Advances in multi‐omics, liquid biopsy, and artificial intelligence (AI)‐driven analytics are redefining early detection and risk stratification strategies for cardiovascular diseases (CVDs).

### 4.4 Multi‐Omics Approaches

Multi‐omics technologies integrate genomics, transcriptomics, proteomics, metabolomics, and epigenomics to identify novel biomarkers at different biological levels. Genomic studies, including genome‐wide association studies (GWAS), have linked genetic variants such as the 9p21 locus to increased cardiovascular risk [54]. Transcriptomic profiling has identified circulating microRNAs (miRNAs) (e.g., miR‐126, miR‐208) as potential early indicators of myocardial infarction (MI) and heart failure. Proteomic techniques utilizing mass spectrometry have discovered growth differentiation factor‐15 (GDF‐15) and suppressor of tumorigenicity 2 (ST2) as promising markers for myocardial stress and fibrosis [54]. Metabolomics has revealed associations between metabolic byproducts like trimethylamine N‐oxide (TMAO) and adverse cardiovascular outcomes, facilitating personalized risk assessment [56].

### 4.5 Liquid Biopsy for Noninvasive Biomarker Detection

Liquid biopsy represents a groundbreaking, minimally invasive technique that enables the detection of circulating biomarkers from blood, urine, or other body fluids, offering a real‐time assessment of cardiovascular disease progression. Unlike traditional tissue biopsies, liquid biopsy allows for repeated sampling, making it a powerful tool for early disease detection and monitoring.

- Circulating Cell‐Free DNA (cfDNA): Myocardial injury leads to the release of fragmented cfDNA into the bloodstream. Studies have demonstrated that cfDNA levels rise before troponin elevation, suggesting it as an early biomarker for acute myocardial infarction (AMI). Additionally, methylation patterns of cfDNA can differentiate between ischemic and nonischemic heart diseases [57].
- Circulating RNA (microRNAs and Long Noncoding RNAs): MicroRNAs (e.g., miR‐1, miR‐133, and miR‐208) are small, noncoding RNAs that regulate gene expression and have been implicated in myocardial injury, hypertrophy, and fibrosis. Long noncoding RNAs (lncRNAs) such as LIPCAR (long intergenic noncoding RNA predicting cardiac remodeling) have been associated with heart failure progression [54, 55, 56, 57, 58, 59, 60].
- Extracellular Vesicles (EVs) and Exosomes: EVs, including exosomes, are lipid bilayer vesicles secreted by various cell types, including cardiomyocytes and endothelial cells. These vesicles carry disease‐specific molecular cargo, such as proteins, lipids, and RNAs, which reflect the pathological state of the cardiovascular system. Exosomal biomarkers have shown potential in diagnosing coronary artery disease (CAD), heart failure, and atrial fibrillation [55].
- Epigenetic Biomarkers (DNA Methylation and Histone Modifications): Alterations in DNA methylation profiles have been linked to atherosclerosis, hypertension, and cardiomyopathies. For instance, hypermethylation of the ATP‐binding cassette transporter A1 (ABCA1) gene has been associated with an increased risk of coronary artery disease [49, 55, 57].
- Circulating Proteins and Metabolites: Beyond nucleic acids, liquid biopsy also enables the detection of novel circulating proteins (e.g., GDF‐15, ST2) and metabolites (e.g., TMAO, branched‐chain amino acids) that offer insights into inflammation, oxidative stress, and metabolic dysregulation in CVD.

### 4.6 Artificial Intelligence (AI) and Machine Learning in Biomarker Discovery

AI and machine learning (ML) are transforming biomarker discovery by integrating vast datasets from multi‐omics studies, imaging, and electronic health records (EHRs) to uncover novel biomarkers with superior predictive accuracy. AI‐powered algorithms enhance pattern recognition, enabling early detection and risk stratification of cardiovascular diseases.

- AI‐Driven Multi‐Omics Data Integration: AI algorithms can process large‐scale genomic, transcriptomic, and proteomic data to identify complex biomarker signatures associated with CVD progression. For example, deep learning models have been used to predict heart failure onset by analyzing genomic variants, protein expression levels, and metabolic profiles in a single framework [61].
- AI‐Based Electrocardiogram (ECG) Analysis: AI‐enhanced ECG interpretation has shown promise in detecting silent atrial fibrillation, left ventricular dysfunction, and early‐stage heart failure with higher sensitivity than traditional ECG‐based methods. By analyzing subtle waveform changes, deep learning models can predict the likelihood of adverse cardiovascular events before clinical symptoms appear [62].
- Machine Learning for Imaging Biomarkers: AI‐driven cardiac imaging techniques, including echocardiography, cardiac MRI, and coronary CT angiography, have revolutionized risk stratification. AI‐based models can quantify myocardial fibrosis, detect subclinical atherosclerosis, and assess left ventricular ejection fraction with greater precision than conventional manual interpretation [61].
- Predictive AI Models for Personalized Risk Assessment: AI tools analyze patient‐specific factors, including genetic predisposition, lifestyle, and biomarker profiles, to develop personalized cardiovascular risk scores. These models continuously learn and improve, enabling real‐time decision‐making and targeted interventions [54, 61, 62, 63].
- Natural Language Processing (NLP) for Biomarker Research: NLP algorithms extract valuable insights from published literature, clinical trial data, and EHRs, accelerating biomarker discovery and validation. This approach enables researchers to rapidly identify novel biomarker candidates from vast unstructured datasets [63].

### 4.7 Clinical Advantages Over Traditional Biomarkers

The integration of novel biomarker discovery techniques, including liquid biopsy, multi‐omics, and AI‐driven analytics, provides several advantages over traditional cardiovascular biomarkers. Firstly, these approaches offer significantly higher sensitivity and specificity, detecting disease at earlier stages with greater precision. Unlike traditional biomarkers such as troponins and BNP, which primarily indicate advanced myocardial injury, circulating cell‐free DNA (cfDNA), microRNAs, and extracellular vesicles (EVs) enable the identification of subclinical cardiovascular dysfunction before irreversible damage occurs [54, 58, 59, 61, 64]. Liquid biopsy techniques allow for noninvasive, real‐time monitoring of disease progression and treatment response, reducing the need for invasive procedures like cardiac catheterization. Additionally, AI‐powered predictive models enhance risk stratification by integrating multi‐omics data, imaging biomarkers, and electronic health records (EHRs), enabling a more personalized approach to cardiovascular care. AI‐driven ECG and imaging analyses provide superior diagnostic accuracy in detecting silent atrial fibrillation, left ventricular dysfunction, and early‐stage heart failure compared to traditional manual interpretation. Furthermore, metabolomic profiling identifies biochemical signatures (e.g., TMAO, branched‐chain amino acids) linked to cardiovascular risk, allowing for individualized therapeutic strategies. The ability of these novel technologies to process vast amounts of patient‐specific data facilitates precision medicine, optimizing preventive and therapeutic interventions [32, 54, 58]. Moreover, AI‐based automation reduces clinician workload, enhances workflow efficiency, and minimizes diagnostic errors. Despite the initial costs associated with implementing these advanced technologies, their long‐term benefits—including earlier disease detection, improved patient outcomes, and reduced healthcare costs due to early intervention far outweigh the limitations. As these innovations continue to evolve, they are expected to become integral components of routine cardiovascular risk assessment and disease management [46, 48].

## 5 Challenges Associated With Biomarker Implementation in Clinical Practice

Biomarkers must undergo rigorous validation to ensure their accuracy and reliability. Validation involves assessing their performance across diverse populations and different clinical contexts. This process involves evaluating sensitivity, specificity, positive and negative predictive values, and receiver operating characteristic (ROC) curves. Reproducibility, the ability to obtain consistent results across different laboratories and platforms, is critical to ensure that the biomarker's performance is consistent regardless of where it is tested [57]. Measurements of biomarkers can vary depending on the assay method, equipment, and laboratory practices. Lack of standardization can lead to discrepancies in results between different laboratories. Establishing standardized protocols and reference materials is essential to minimize variability and ensure that biomarker results are comparable across different settings. Biomarkers should provide meaningful information that influences clinical decision‐making and patient outcomes. Simply demonstrating a correlation between a biomarker and a disease is not sufficient. Clinical utility must be demonstrated through prospective studies that show the biomarker's ability to improve patient outcomes, guide treatment decisions, or predict disease progression beyond traditional risk factors [58]. Some biomarker tests can be expensive due to the need for specialized equipment, reagents, and expertise. Ensuring that biomarker testing is affordable and accessible to a wide range of patients is crucial for equitable healthcare delivery. High costs can limit the adoption of biomarkers, especially in resource‐constrained settings [40, 50, 65]. For biomarker implementation to be successful, the testing process should seamlessly integrate into clinical workflows. If biomarker testing is time‐consuming, requires additional appointments, or disrupts the flow of patient care, healthcare providers may be less inclined to use them routinely. Interpreting biomarker results can be complex, especially in the context of risk stratification. Determining appropriate cutoff values for identifying different risk categories requires careful consideration and validation. Overly simplistic interpretation can lead to misclassification of patients' risk levels. Some biomarkers may exhibit variability over time due to factors like lifestyle changes, medications, or disease progression. Longitudinal tracking is necessary to capture trends and assess changes in risk over time. However, this requires repeated testing, which can be cumbersome for both patients and healthcare providers.

### 5.1 Regulatory and Ethical Aspects in Biomarker Development

Rigorous assessment of safety, effectiveness, and clinical utility is required before biomarkers can be approved for diagnostic or prognostic purposes. The regulatory process ensures that biomarkers meet stringent standards for patient safety and accurate clinical use. Biomarker research raises ethical concerns about patient consent, privacy, and data security [64]. Researchers must ensure that patients understand the purpose of biomarker testing, potential risks, and benefits. Data privacy must be maintained throughout the testing, analysis, and reporting stages to protect patients' sensitive information [64]. The development and application of biomarkers for diagnostic and prognostic purposes require rigorous evaluation of safety, effectiveness, and clinical utility. Regulatory processes ensure that biomarkers meet stringent standards to safeguard patient safety and ensure accurate clinical use. Ethical considerations in biomarker research include obtaining informed consent from participants, ensuring they understand the purpose, risks, and benefits of testing, and safeguarding data privacy throughout testing and analysis stages. Transparency in data sharing practices is essential to balance research collaboration with patient privacy and regulatory compliance. Managing conflicts of interest between researchers and industry partners is crucial to maintain the integrity of biomarker development and prioritize patient welfare. Ethical concerns also encompass ensuring equitable access to biomarker testing across diverse populations, addressing potential disparities in healthcare access and affordability [66, 67, 68, 69].

## 6 Future Directions and Opportunities for Biomarker Advancements in Cardiovascular Diseases

Advancements in biomarkers for cardiovascular diseases hold promise for transforming risk assessment, diagnosis, treatment, and patient outcomes. Integrating state‐of‐the‐art technologies such as AI and personalized medicine approaches will enhance accuracy in risk assessment and early detection. Genomics, proteomics, and metabolomics offer opportunities to develop tailored risk scores by considering genetic predispositions, molecular profiles, and environmental influences. Combining data from these omics disciplines can unveil new biomarkers and disease signatures for precise risk prediction. AI and machine learning algorithms analyze complex biomarker profiles, improving predictive capabilities and aiding clinical decision‐making. Wearable devices and mobile health apps provide real‐time physiological data, complementing traditional biomarkers for a comprehensive view of cardiovascular health. Personalized therapeutic interventions guided by biomarkers ensure optimal treatment outcomes while minimizing risks. Exploring microbiome biomarkers and advancing liquid biopsy techniques offer noninvasive insights into disease mechanisms and potential monitoring solutions. Continuous biomarker monitoring over time informs disease progression and treatment efficacy, necessitating robust validation through large‐scale clinical trials for widespread clinical adoption.

## Author Contributions

**Abubakar Nazir:** conceptualization, writing – original draft, writing – review and editing, visualization, project administration. **Awais Nazir:** writing – original draft, writing – review and editing. **Usama Afzaal:** writing – original draft, writing – review and editing. **Shafaq Aman:** writing – original draft, writing – review and editing. **Safi ur Rehman Sadiq:** writing – original draft, writing – review and editing. **Ozoemena Z. Akah:** writing – original draft, writing – review and editing. **Muhammad Shah Wali Jamal:** writing – original draft, writing – review and editing. **Syed Zawahir Hassan:** writing – original draft, writing – review and editing.

## Ethics Statement

The authors have nothing to report.

## Conflicts of Interest

The authors declare no conflicts of interest.

## Transparency Statement

The lead author Abubakar Nazir affirms that this manuscript is an honest, accurate, and transparent account of the study being reported; that no important aspects of the study have been omitted; and that any discrepancies from the study as planned (and, if relevant, registered) have been explained.

## Data Availability

The authors have nothing to report.
